# Improving prostate cancer detection in veterans through the development of a clinical decision rule for prostate biopsy

**DOI:** 10.1186/1471-2490-13-6

**Published:** 2013-01-29

**Authors:** Owen T Hill, Thomas J Mason, Skai W Schwartz, Philip R Foulis

**Affiliations:** 1 Injury Epidemiology Research Section, Military Performance Division, United States Army Institute of Environmental Medicine, Natick, MA, USA; 2 Department of Environmental and Occupational Health, College of Public Health, University of South Florida, Tampa, FL, USA; 3 Department of Epidemiology and Biostatistics, College of Public Health, University of South Florida, Tampa, FL, USA; 4 Department of Pathology and Laboratory Medicine, James A. Haley Veteran’s Administration, Tampa, FL, USA

## Abstract

**Background:**

We sought to improve prostate cancer (PC) detection through developing a prostate biopsy clinical decision rule (PBCDR), based on an elevated PSA and laboratory biomarkers. This decision rule could be used after initial PC screening, providing the patient and clinician information to consider prior to biopsy.

**Methods:**

This case–control study evaluated men from the Tampa, Florida, James A. Haley (JH) Veteran’s Administration (VA) (N = 1,378), from January 1, 1998, through April 15, 2005. To assess the PBCDR we did all of the following: 1) Identified biomarkers that are related to PC and have the capability of improving the efficiency of PC screening; 2) Developed statistical models to determine which can best predict the probability of PC; 3) Compared each potential model to PSA alone using Receiver Operator Characteristic (ROC) curves, to evaluate for improved overall effectiveness in PC detection and reduction in (negative) biopsies; and 4) Evaluated dose–response relationships between specified lab biomarkers (surrogates for extra-prostatic disease development) and PC progression.

**Results:**

The following biomarkers were related to PC: hemoglobin (HGB) (OR = 1.42 95% CI 1.27, 1.59); red blood cell (RBC) count (OR = 2.52 95% CI 1.67, 3.78); PSA (OR = 1.04 95% CI 1.03, 1.05); and, creatinine (OR = 1.55 95% CI 1.12, 2.15). Comparing all PC stages versus non-cancerous conditions, the ROC curve area under the curve (AUC) enlarged (increasing the probability of correctly classifying PC): PSA (alone) 0.59 (95% CI 0.55, 0.61); PBCDR model 0.68 (95% CI 0.65, 0.71), and the positive predictive value (PPV) increased: PSA 44.7%; PBCDR model 61.8%. Comparing PC (stages II, III, IV) vs. other, the ROC AUC increased: PSA (alone) 0.63 (95% CI 0.58, 0.66); PBCDR model 0.72 (95% CI 0.68, 0.75), and the PPV increased: 20.6% (PSA); PBCDR model 55.3%.

**Conclusions:**

These results suggest evaluating certain common biomarkers in conjunction with PSA may improve PC prediction prior to biopsy. Moreover, these biomarkers may be more helpful in detecting clinically relevant PC. Follow-up studies should begin with replicating the study on different U.S. VA patients involving multiple practices.

## Background

The number of men who undergo prostate biopsies to rule out prostate cancer (PC) increases annually (estimated at over one million per year) [1]. This is in large part a result of elevated serum prostate specific antigen (PSA) values identified during routine PC screening [2,3]. Debate over the appropriateness of PC screening continues [1-7]. In addition, there is considerable controversy over the course one should take upon detecting PSA elevations [2-5]. Moreover, it is inconclusive whether early PC detection results in lower morbidity and mortality for the men identified [3-6].

PC screening has been fraught with controversy and the overtreatment of low-risk PC is considered a major public health problem [8,9]. The U.S. Preventive Services Task Force (USPSTF), National Cancer Institute (NCI), World Health Organization (WHO), and other international agencies do not recommend PC screening [7]. Conversely, the American Cancer Society (ACS), American College of Radiology (ACR), and the American Urological Association (AUA) recommend screening men above the age of 50 with a routine serum PSA and digital rectal exam (DRE) [7]. Proponents for routine PC screening argue that it is a valuable early detection tool as it can identify PC in asymptomatic men prior to clinical presentation. In theory, earlier identified PC should be at a less advanced stage, which implies a more treatable state. This is controversial, as PC is heterogeneous, in some cases indolent (never becoming clinically evident), while in other cases PC can be aggressive, rapidly advancing from a pre-clinical state to distant metastases [8].

An easy to implement screening tool that detects ‘aggressive’ PC is needed. The primary goal of PC screening is to detect cancer before it is too advanced for treatment, and to bypass the tumors that are not destined to shorten a man’s life [9]. With that stated, delineation between different types of PC is difficult, but of paramount importance. PSA research over the last two decades has improved our ability to identify PC [2,3,8,10]. However; this has resulted in needless biopsies and treatments and is a reason for the protracted debate over the PSA utility as a PC screening tool [3].

Despite a wealth of published literature that has evaluated PSA and argued against its use, it remains a mainstay for patients and clinicians. Prior attempts at improving PC screening have focused on replacing PSA with a new test. Tools such as PSA velocity, PSA density, Free/Total PSA ratio, and PCA3 testing have all shown promise for improving PC screening, but difficult implementation and a lack of universal acceptance among clinicians have hindered incorporation into daily clinical practice [2,3,5]. Clearly, PC screening is in need of improvement. Therefore, the purpose of this investigation was to improve the efficiency of PC detection through the development of a novel clinical decision rule. We investigated and propose the prostate biopsy clinical decision rule (PBCDR) that implements both elevated PSA and readily available laboratory biomarkers. If accurate, the PBCDR could be used as an advanced screening tool after PSA as it provides additional clinically significant information to assist the clinician and patient in reaching a decision regarding the urgency of a prostate biopsy.

To develop and validate this advanced screening tool we did all of the following: (1) Identified biomarkers that are related to PC and have the capability of improving the efficiency of PC screening; (2) Developed statistical models to determine which can best predict the probability of PC; (3) Compared each potential model to the current screening tool (PSA only) using ROC curves, to evaluate for improved overall effectiveness in PC detection and reduction in (negative) biopsies; (4) Evaluated dose–response relationships between specified lab biomarkers (surrogates for extra-prostatic disease development) and PC progression. If present, the degree of change between the reference ‘normal’ value and the observed lab value could provide valuable insight for differentiating between indolent and clinically relevant PC.

## Methods

### Population, inclusion/exclusion criteria, and data sources

This study was a case–control analysis, evaluating 1,378 prior military servicemen (40–90 years of age), from the James A. Haley Veteran’s Administration (VA) hospital from January 1, 1998 through April 15, 2005 who had undergone prostate biopsy. Only patients with a PSA value of 4 ng/dL (or higher), with laboratory data obtained at the time of the PSA sample, and an initial diagnosis of PC, prostatic interstitial neoplasm (PIN), benign prostatic hypertrophy (BPH), or prostatitis were included in the analysis. Patients with history of prior genital urinary malignancy, and those with inadequate biopsy specimens were excluded from analysis. Subjects meeting inclusion criteria were classified into one of four ‘histology’ groups. The cases consist of biopsy-confirmed PC. The non-PC cases were classified into three groups: (1) PIN, (2) BPH, or (3) prostatitis. Biopsies with isolated atypical small acinar proliferation were included in the PIN group. This study was approved by the Institutional Review Board at the University of South Florida and James A. Haley VA hospital.

### Case identification/data collection methods

A case of PC is defined by prostate tissue that demonstrates cells of adenocarcinoma on histologic evaluation [11]. With that, identification of study subjects was accomplished through searching the Anatomic Pathology portion of VisTa (Systematized Nomenclature of Medicine (SNOMED) finalized accession logs) to find all cases coded as 'prostate disease' (SNOMED codes: 77220, 77103, 77102, 77101, 77110, 77105, 77350, 77230, 77210, 77300, 77200, 77240, 77104, 77100, 77000, 77900, 77250). SNOMED is a classification system of the College of American Pathologists, and is the standard tool used by pathologists to create, share, and retrieve pathology information. The patient’s identification number, date of the specimen, diagnosis text code, and accompanying narrative text description were captured through this VISTA search. The collection of both the diagnosis text code and accompanying text description was performed intentionally as a way to validate the histologic diagnosis. For instance, if the diagnosis code was ‘adenocarcinoma of the prostate’ (SNOMED code 77220); the corresponding narrative text description would provide the same diagnosis. The goal of this initial search was to capture all prostate related cases; hence the large number of SNOMED codes used.

The James Haley VA electronic medical records (EMR) were then accessed for all of the potential PC cases to validate the diagnosis. This was accomplished by confirming the International Classification of Diseases, ninth revision (ICD–9) PC codes and capturing basic demographic, laboratory biomarkers, and any previous pathology results.

Data reduction strategies were employed which ultimately left 1,378 participants available (from 2,575 potential subjects) for statistical analysis. Over 500 prostate biopsies (541) did not meet inclusion criteria because they were performed on men who did not have a PSA test value of 4.0 ng/dL or greater. Typical situations that would result in this scenario include biopsies secondary to suspicious DRE or Transrectal ultrasound (TRUS), or other reasons (e.g. positive family history). There were 321 biopsies identified as “repeat biopsies”, which were then dropped from the study. Next, biopsies performed as a result of other genital urinary malignancies (i.e. bladder, renal, ureter, and penile cancer) (N=260) were excluded. It is important to note that the specific prompting for all prostate biopsies was confirmed through a detailed record review of each prostate biopsy report within the VISTA system. These notes were in narrative form and were validated by confirming the ICD-9 code for that event. Lastly, participants without a full complement of laboratory biomarkers were excluded from the study (N=75). Thus our final analysis was based on 1,378 patients.

### Outcomes - PC classification

PC classification was based on Stage and Gleason sum for each patient with histologic confirmation of PC. The Stages were categorized as follows: Stage I for non-palpable, prostate contained cancer (analogous to T1 non-palpable PC); Stage II for palpable prostate contained cancer (analogous to T2 palpable PC); Stage III for locally spread PC (analogous to T3/T4 PC); and Stage IV for metastatic PC (analogous to M1 PC). To determine the Gleason sum, the two most prominent areas of PC activity (as identified by the evaluating pathologist on histologic examination) were identified. Each prominent area was given a score of 1–5, with 1 being well differentiated, and 5 being poorly differentiated (implying a more aggressive appearance). The two scores are added together, and this number was the recorded Gleason sum for all subsequent analyses.

### Statistical analysis

Univariate, bivariate, logistic regression, linear regression, and receiver operator characteristic (ROC) curves were utilized. Data were analyzed utilizing SAS statistical software. Descriptive analysis allowed for careful review of data frequencies, measures of central tendency, and distribution shapes. Bivariate analysis tested all identified biomarkers to determine the statistical significance and degree of correlation between these independent variables and PC. Multivariate logistic regression was utilized to evaluate which statistical model can best predict the probability of PC.

We first defined a “Case” as any stage of PC and a “Control” as any non-cancerous prostate condition (PIN, BPH, and prostatitis). In a secondary analysis, we redefined a “Case” as PC stages (II, III, IV) and “Control” as stage I PC, or any non-cancerous prostate condition. Our reasoning was that as Stage 1 cancer is localized, it may be unlikely to progress to advanced disease.

A complete model (including age, ethnicity, biomarkers, and any interaction terms) was established initially. Exclusion of each covariate was performed, looking for a change in the overall – 2 Log Likelihood Ratio. A potential covariate was permanently removed from the model development process if there was no effect on the overall – 2 Log Likelihood value. This lack of change indicates the variable is not contributing to the prediction of the outcome, and conversely, if there was a change of statistical significance, the variable was included in the final model. To test for differences between the full model and the final model, evaluation of the likelihood ratio p-value was performed, in which a value greater than 0.05 indicated a satisfactory fit of the smaller model. Regression diagnostic techniques were employed to increase the reliability of the data.

ROC curves were used to address which statistical models could best predict the probability of PC and if a dose–response relationship existed between specified lab biomarkers and PC progression. ROC curves are graphical tools, which plot the sensitivity vs. 1- the specificity for a binary classification system (as a function of changes in the cut-off value threshold). This analysis technique was used to judge the validity of proposed model. Mean and median values of all significant biomarkers were determined for each PC stage subset to determine if a gradient exists between advancing PC severity and laboratory biomarkers.

## Results

Table 1 outlines the demographics of the study participants. The mean age of the subjects was 68 years (SD=8.5). Most of the men had either prostatitis, BPH, or stage I PC (79.4%). Stage II, III and IV PC accounted for 20.6% of all diagnoses. The study population was largely Caucasian (study total=70.75%, PC=69.4%, PIN=71.73%, BPH=74.82%, prostatitis=69.29%). Age at diagnosis (PC stage I group=68.37, PC stage II group=69.73, PC stage III group=67.73, stage IV group 67.88, PIN group=67.75, BPH group=67.83, prostatitis group=68.17) was comparable across all diagnosis groups.

**Table 1:** Demographics of study particpants by prostate biopsy results

	**Prostatitis**	**BPH**	**PIN**	**PC stage I**	**PC stage II**	**PC stage III**	**PC stage IV**
	**N=342**	**N=282**	**N=138**	**N=332**	**N=220**	**N=48**	**N=16**
Ethnicity*							
Caucasian	237(69%)	211(75%)	99 (72%)	238 (72%)	149 (68%)	31(65%)	10 (63%)
Black	18 (5%)	19 (7%)	13 (9%)	34 (10%)	22 (10%)	7 (15%)	4 (25%)
Hispanic	22 (6%)	14 (5%)	7 (5%)	15 (5%)	11 (5%)	1 (2%)	1 (6%)
Other	65 (20%)	38 (14%)	19 (14%)	45 (13%)	38 (17%)	9 (18%)	1 (6%)
Mean Age**	68.17	67.83	67.75	68.37	69.73	67.73	67.88
SD Age	8.54	8.19	8.49	8.99	9.36	9.39	10.6

* P-value < 0.05.

** Not statistically significant.

Table 2 outlines the mean value and standard deviation of all continuous laboratory biomarkers by the prostate biopsy results. The mean values of albumin, hemoglobin (HGB), RBC count, creatinine, and folate all decreased as the stage of PC increased. Conversely, the mean value of PSA increased as PC stage increased. No trends were observed for mean values of mean corpuscular volume **(**MCV), BUN, platelet count, WBC, LDH, and bilirubin. Trend significance tests revealed that as the prostate biopsy result increased from prostatitis to stage IV PC, the mean value of HGB, creatinine, PSA, and BUN varied from reference normal (p < 0.05). No trends were observed for the lab biomarkers RBC, MCV, albumin, WBC, bilirubin, and platelet count.

**Table 2:** Mean value and SD of Lab biomarkers by Prostate biopsy results

	**Prostatitis**	**BPH**	**PIN**	**PC stage I**	**PC stage II**	**PC stage III**	**PC stage IV**
	**N=342**	**N=282**	**N=138**	**N=332**	**N=220**	**N=48**	**N=16**
*Albumin*							
Mean	4	3.98	4.09	4.04	4	3.95	3.9
*SD*	0.38	0.37	0.37	0.38	0.42	0.39	0.42
*HGB* *							
Mean	14.27	14.24	14.3	14.17	13.7	12.59	12.84
SD	1.54	1.76	1.68	1.66	1.87	2.2	14.08
*RBC*							
Mean	4.67	4.65	4.76	4.69	4.63	4.58	4.5
SD	0.46	0.52	0.52	0.5	0.57	0.44	0.67
*Creatinine*							
Mean	1.25	1.16	1.2	1.16	1.15	1.14	1.13
SD	0.7	0.32	0.35	0.56	0.34	0.39	0.2
PSA*							
Mean	8.06	7.99	7.67	9.7	14.12	21.85	44.03
SD	6.74	11.2	4.58	11.66	29.27	30.21	48.71
*MCV*							
Mean	90.78	91.13	90.09	91.17	90.98	90.23	92.24
SD	4.65	5.35	5.79	5.05	6.11	4.18	4.99
*Bilirubin*							
Mean	0.66	0.65	0.6	0.66	0.67	0.65	0.6
SD	0.4	0.3	0.25	0.35	0.35	0.34	0.3
BUN*							
Mean	17.98	17.6	17.23	16.53	17.4	16	15.97
SD	6.93	7.9	6.5	6.56	6.73	6.74	4.54
*Platlet*							
Mean	230.45	227.63	240.81	226.54	232.5	234.3	235.44
SD	59.16	61.56	84.31	62.26	69.25	68.07	50.76
*WBC*							
Mean	7.24	7.11	7.42	7.19	7.13	10.41	7.37
SD	2.11	2.17	3.24	2.42	2.08	17.78	1.84
*Folate*							
Mean	12.93	12.56	12.31	13.37	11.92	10.71	10.03
SD	5.44	5.78	5.29	5.25	5.43	5.44	4.99
*LDH*							
Mean	426.91	424.78	371.84	404.6	406.41	392.11	393.33
SD	375.09	144.84	185.54	155.38	158.19	170.98	73.76

*Trend significance (P<0.05).

Crude Odds Ratios (with 95% CI) for the association of each laboratory biomarker with PC are presented in two ways:

**Primary analysis:** all PC stages vs. non-cancerous prostate conditions

**Secondary analysis:** PC (stages II, III, IV) vs. (PC stage I, PIN, BPH, and prostatitis)

### Primary analysis

In this analysis, Hemoglobin, PSA, and serum BUN were related significantly to PC positive cases (when compared to non-cancerous prostate conditions) (Table 3).

**Table 3:** Odds ratio and 95% CI for method 1: PC (all stages) vs. non-cancerous conditions, per laboratory unit

**Covariates included**	**Odds ratio**	**OR 95% confidence interval**
HGB *	0.872	(.821-.927)
Age	1.01	(.99-1.02)
PSA*	1.04	(1.02-1.06)
Hematuria	0.953	(0.86-1.06)
Proteinuria	0.943	(0.83-1.07)
Albumin	1.03	(0.78-1.36)
Creatinine	0.762	(0.76-1.01)
MCV	1.01	(0.99-1.03)
PLT	1	(0.99-1.00)
RBC	0.918	(0.75-1.13)
Total Bilirubin	1.14	(0.84-1.55)
BUN*	0.98	(0.96-0.99)
WBC	1.01	(0.98-1.05)

*P < 0.05.

### Secondary analysis

When the stage I PC subjects were placed in the comparison group (leaving stage II, III, and IV PC subjects as the ‘cases’), hemoglobin, age, PSA, hematuria, and RBC count demonstrated statistically significant relationships with the PC positive cases (when compared to the comparison group: stage I PC, PIN, BPH, and prostatitis) (Table 4).

**Table 4:** Odds ratio and 95% CI for method2: PC (stage II, III, IV) vs. other (PC stage I, PIN, BPH, prostatitis), per laboratory unit

**Covariates included**	**Odds ratio**	**OR 95% confidence interval**
HGB *	0.79	(.738-.851)
AGE*	1.02	(1.01-1.03)
PSA*	1.04	(1.03-1.05)
Hematuria*	1.23	(1.10-1.38)
Proteinuria	1.16	(1.00-1.34)
Albumin	0.77	(0.55-1.08)
Creatinine	0.76	(0.52-1.12)
MCV	1	(0.97-1.03)
PLT	1	(0.99-1.00)
RBC*	0.76	(0.59-0.98)
Total Bilirubin	1.15	(0.80-1.66)
BUN	0.99	(0.98-1.01)
WBC	1.03	(0.99-1.06)

*P < 0.05.

Table 5 summarizes laboratory biomarkers, which demonstrate a statistically significant relationship with PC.

**Table 5:** Summary table of independent variables that demonstrate statistically significant relations with PC (by analysis methods 1–2)

**Independent variable**	**Method 1**	**Method 2**
Hemoglobin (HGB)	**	**
RBC count		*
BUN	*	
Hematuria		*
PSA	**	**
Age		*

*P <0.05, ** P <0.001.

Table 6 outlines the PPV of PSA (> 4 ng/dL) alone for PC detection. This PPV was evaluated as method 1 and 2 (described above).

**Table 6:** Positive predictive value of PSA > 4 ng/dL

**Comparison groups**	**Method 1**	**Method 2**
PC Cases/Total biopsies	616/1,378	284/1,378
Positive Predictive Value	44.70%	20.60%

Method 1: PC (all stages) vs. non-cancerous conditions (PIN, BPH, prostatitis).

Method 2: PC (stage II, III, IV) vs. other (stage I PC, PIN, BPH, prostatitis).

The positive predictive value was decreased significantly when stage I PC was not considered a case. In particular, the PPV decreased by 24.1% when the stage I PC group was considered in the comparison group.

Multiple models were run, individually excluding each covariate, to assess the change of the −2 Log Likelihood value and the C-statistic. Analyses were terminated when the model with the lowest −2 Log Likelihood value and highest C-statistic was determined for each of the two analysis methods (Tables 7 and 8).

**Table 7:** Best fit logistical regression for method 1: risk of PC with lab biomarkers and 95% CI, per laboratory unit method 1: PC (all stages) vs. non-cancerous conditions (PIN, BPH, prostatitis)

**Parameter**	**ML Est.**	**SE**	**OR**	**95% CI**	**C stat**	**−2 LL**
Intercept	−7.9494	1.88	xx	xx	0.68	1777.339
HGB*	−0.3519	0.06	0.70	(0.63-.79)		
RBC*	−0.9227	0.21	0.40	(0.26-0.60)		
Hematuria*	−0.2874	0.15	0.75	(0.56-1.01)		
Creatinine*	−0.4393	0.17	0.65	(0.47-0.89)		
Black*	0.6336	0.21	1.89	(1.25-2.90)		
PSA*	0.0408	0.08	1.04	(1.03-1.06)		
AGE*	0.0196	0.01	1.02	(1.01-1.03)		
MCV*	0.0663	0.02	1.07	(1.04-1.10)		
Albumin	0.2871	0.16	1.33	(0.98-1.82)		

*P < 0.05.

**Table 8:** Best fit logistical regression model for method 2: risk and 95% CI for PC with lab biomarkers, per laboratory unit

**Parameter**	**ML Est.**	**SE**	**OR**	**95% CI**	**C Stat**	**−2 LL**
Intercept	−5.1041	xx	xx	xx	0.713	1276.366
HGB*	−0.3784	0.05	0.69	(0.62, 0.76)		
RBC*	−0.7641	0.21	0.46	(0.30, 0.70)		
Creatinine*	−0.6069	0.23	0.55	(0.35, 0.85)		
PSA *	0.0325	0.01	1.03	(1.02, 1.05)		
Age*	0.0183	0.01	1.02	(1.01, 1.04)		
MCV*	0.0488	0.02	1.05	(1.02, 1.08)		
Black	0.3612	0.24	1.44	(0.90, 2.31)		

*P <0.001.

**Figure 1:**
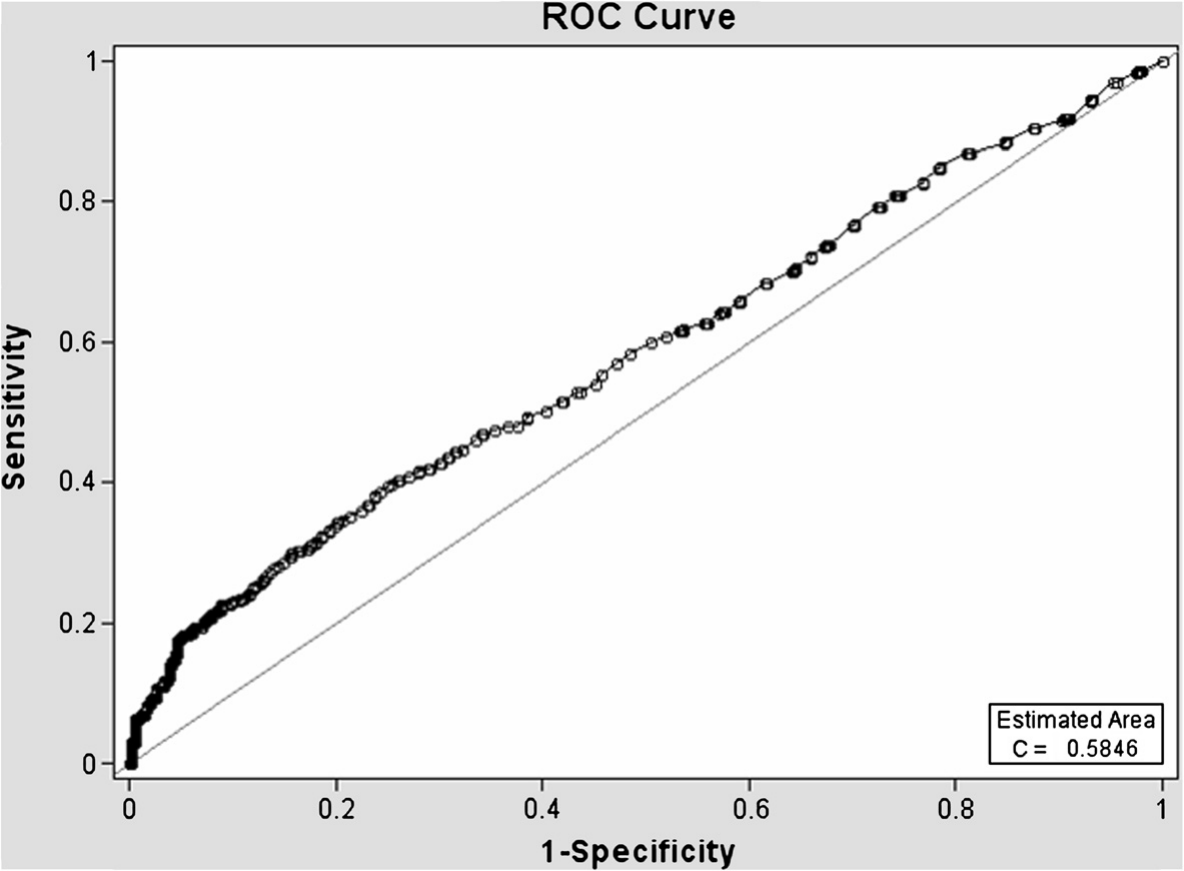
Diagnostic statistics = ROC curve of PSA Alone - Method 1.

**Figure 2:**
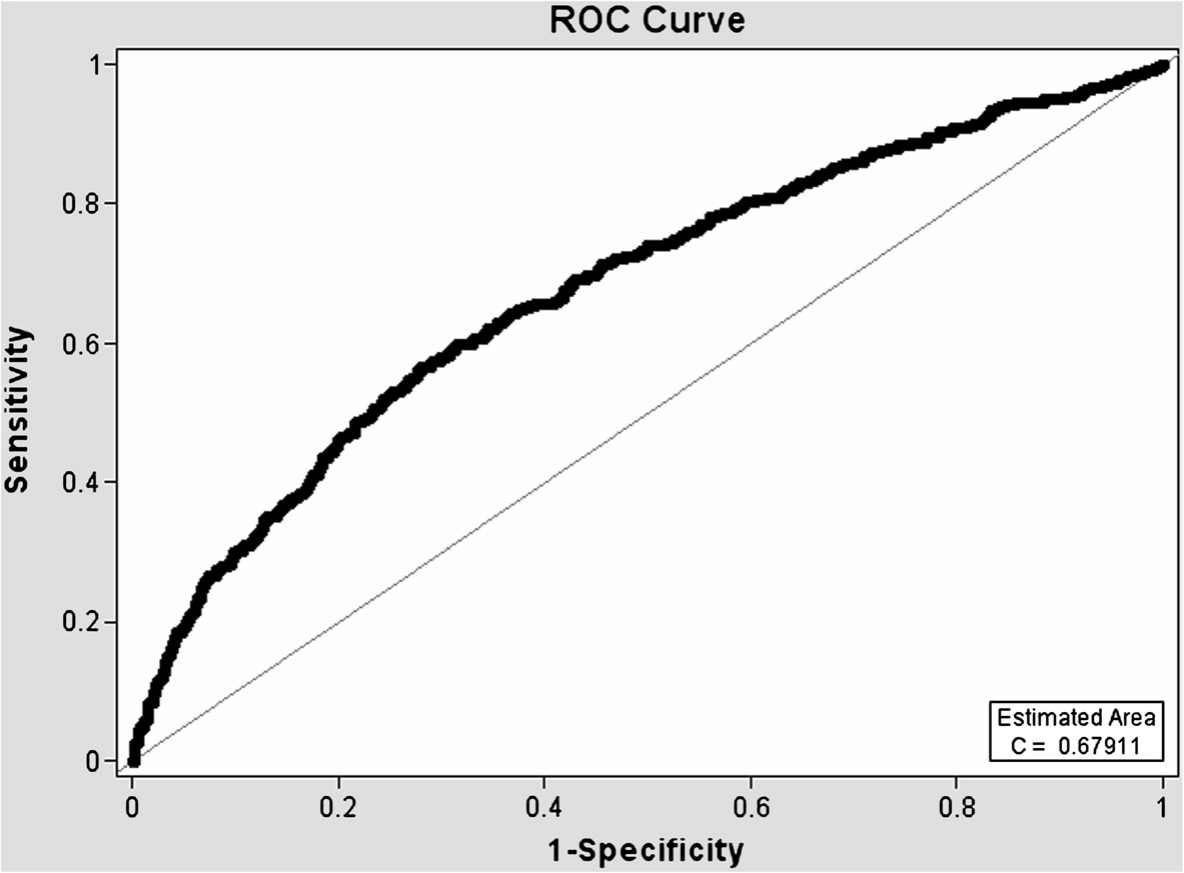
Diagnostic statistics = ROC curve of PSA + lab biomarkers - Method 1.

**Figure 3:**
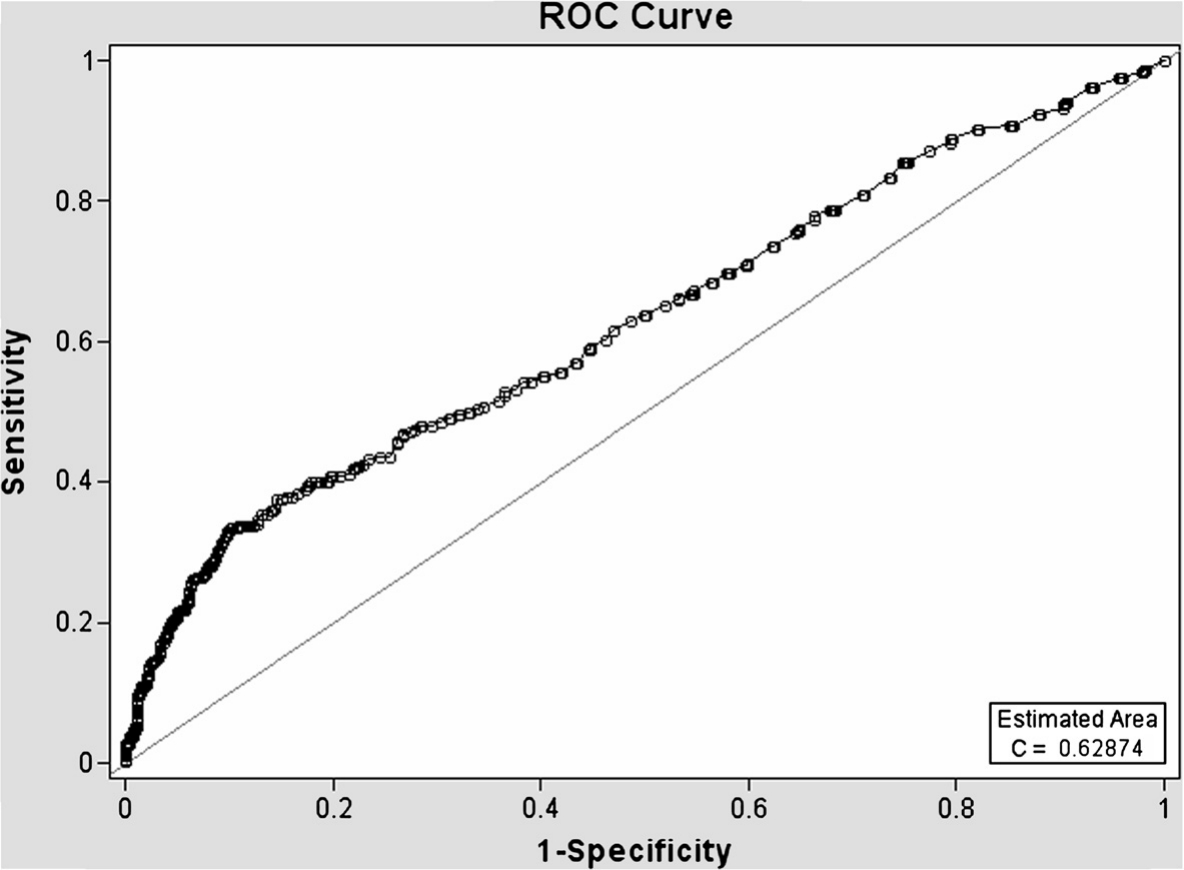
Diagnostic statistics = ROC curve of PSA Alone - Method 2.

**Figure 4:**
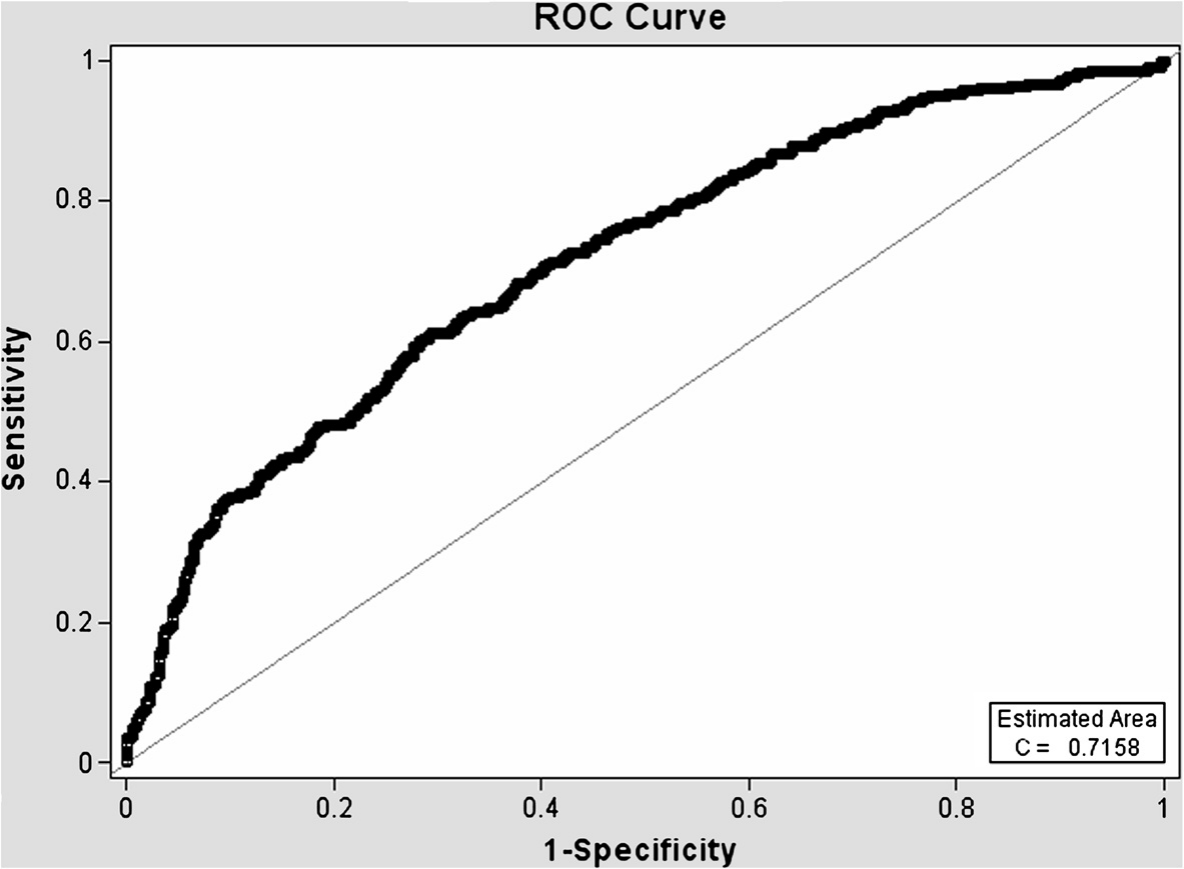
Diagnostic statistics = ROC curve of PSA + lab biomarkers - Method 2.

ROC curves (Figures 1, 2, 3 and 4) demonstrate the validity of analysis method 1 and 2, respectively and the difference between the existing PC screening test (PSA alone) and the PBCDR. Confidence intervals between PSA alone and PBCDR models (PSA + lab biomarkers) did not overlap and were statistically significantly different. **The ROC AUC:** Method 1 **PSA alone 0.59, (95% CI 0.55, 0.61) to PBCDR (PSA+ significant lab biomarkers) 0.68 (95% CI 0.65, 0.71);** Method 2 **PSA alone 0.63, (95% CI 0.58, 0.66) to PBCDR (PSA+ significant lab biomarkers) 0.72 (95% CI 0.68, 0.75).**

To determine the ideal cut-points for recommending prostate biopsy, four different cut-points where chosen, each providing either increased sensitivity or specificity. The cut-points are presented in four ways:

• *Cut-point 1* – The maximum likelihood ratio. This was determined by dividing the sensitivity by 1- the specificity (SEN/1-SPC), thus maximizing the quotient.

• *Cut point 2* – The probability that yielded a sensitivity of approximately 90% with the highest corresponding specificity.

• *Cut point 3* – The probability that yielded a sensitivity of approximately 80% with the highest corresponding specificity.

• *Cut point 4* – The probability that yielded a specificity of approximately 80% with the highest corresponding sensitivity (Table 9).

**Table 9:** Sensitivity, specificity, PPV, and NPV for probability cut-off points

	**Method 1**	**Method 2**
Cut-point 1 (MLE)	Probability .45	Probability .41
Sensitivity	52.1 %	18.3 %
Specificity	74.0 %	96.2 %
PPV	61.8 %	55.3 %
NPV	65.7%	81.9 %
Cut-point 2 (Sen. 90%)	Probability .33	Probability .13
Sensitivity	90.9 %	89.8 %
Specificity	17.6 %	28.0 %
PPV	47.1 %	20.6 %
NPV	70.5 %	91.3 %
Cut-point 3 (Sen. 80%)	Probability .37	Probability .15
Sensitivity	80.5 %	78.2 %
Specificity	37.1 %	45.0 %
PPV	50.9 %	28.7 %
NPV	70.2 %	88.8 %
Cut-point 4 (Spc. 80%)	Probability .48	Probability .23
Sensitivity	39.9 %	45.8 %
Specificity	81.4 %	79.5 %
PPV	63.4 %	36.7 %
NPV	62.6 %	85.0 %

Table 10 summarizes the PPV between PSA alone and the PBCDR. Within each analysis method the PBCDR has increased PPV percentages, respectively.

**Table 10:** Comparison of PPV between PSA (> 4ng/dL) and cut-points 1–4 by analysis method

	**Method 1**	**Method 2**
Crude PPV	44.7 %	20.6 %
Cut-point 1	61.8 %	55.3 %
Cut-point 2	47.1 %	20.6%
Cut-point 3	50.9 %	28.7%
Cut-point 4	63.4 %	36.7%

To evaluate our hypothesis that there exists a gradient between specified lab biomarkers and increasing PC stage, the mean values of each specified laboratory biomarker (with accompanying 95% CI) was determined for each PC stage. In agreement with our hypothesis, we observed a change in the mean value of the biomarkers, HGB, RBC, Albumin and PSA, away from the normal reference levels as the PC stage increased (Table 11).

**Table 11:** Mean value, SD, and 95% CI of lab biomarkers by PC stage

	**PC Stage A**	**PC Stage B**	**PC Stage C**	**PC Stage D**
	**N=332**	**N=220**	**N=48**	**N=16**
Albumin*				
Mean	4.04	4.00	3.95	3.90
SD	0.38	0.42	0.39	0.42
95%CI	(4.00, 4.08)	(3.96, 4.05)	(3.84, 4.06)	(3.69, 4.11)
HGB*				
Mean	14.17	13.70	12.59	12.84
SD	1.66	1.87	2.20	14.08
95%CI	(14.00, 14.35)	(13.45, 13.95)	(11.97, 13.21)	(5.94, 19.74)
*RBC* *				
Mean	4.69	4.63	4.58	4.50
SD	0.50	0.57	0.44	0.67
95%CI	(4.64, 4.74)	(4.55, 4.71)	(4.46, 4.70)	(4.17, 4.83)
*Creatinine*				
Mean	1.16	1.15	1.14	1.13
SD	0.56	0.34	0.39	0.20
95%CI	(1.10, 1.22)	(1.11, 1.20)	(1.03, 1.25)	(1.03, 1.23)
PSA*				
Mean	9.70	14.12	21.85	44.03
SD	11.66	29.27	30.21	48.71
95%CI	(8.45, 10.95)	(10.25, 17.99)	(13.31, 30.39)	(20.16, 67.90)
*MCV*				
Mean	91.17	90.98	90.23	92.24
SD	5.05	6.11	4.18	4.99
95%CI	(90.63, 91.71)	(90.17, 91.79)	(89.05, 91.41)	(89.79, 94.69)
*Bilirubin*				
Mean	0.66	0.67	0.65	0.60
SD	0.35	0.35	0.34	0.30
95%CI	(0.28, 1.04)	(0.62, 0.72)	(0.60, 0.70)	(0.45, 0.75)
*BUN*				
Mean	16.53	17.40	16.00	15.97
SD	6.56	6.73	6.74	4.54
95%CI	(15.82, 17.24)	(16.51, 18.29)	(17.91, 14.09)	(13.74, 18.20)
*Platlet*				
Mean	226.54	232.50	234.30	235.44
SD	62.26	69.25	68.07	50.76
95%CI	(219.8, 233.2)	(223.4, 241.7)	(215.1, 253.6)	(210.6, 260.3)
*WBC*				
Mean	7.19	7.13	10.41	7.37
SD	2.42	2.08	1.78	1.84
95%CI	(6.93, 7.45)	(6.86, 7.40)	(9.91, 10.91)	(6.47, 8.27)

* P <0.05.

A trend analysis was performed to determine whether or not the change seen with lab biomarkers HGB, RBC, Albumin, and PSA was statistically significant. Models were analyzed by first coding each continuous laboratory biomarkers as the criterion variable, with the PC stage coded as the predictor. The model results suggest that PC stage is a significant statistical predictor for gradient changes in HGB, RBC count, Albumin, and PSA.

A case example providing a glimpse of the real world application of the proposed PBCDR is outlined below (Table 12).

**Table 12:** Case example utilizing method 2, cut-point 1

**Prob. .41/1-.41 = -0.16**	**Patients value**	**Parameter estimate**	Pt. value *Parameter estimate	**Intercept**
HGB	15.2	-0.378	-5.74	-5.10
RBC	4.50	-0.764	-3.44	
Black	(Yes=1) 0	0.361	0.00	
Age	60	0.018	1.08	
PSA	4.3	0.033	0.129	
Creatinine	1.65	-0.601	-0.99	
MCV	90.00	0.050	4.5	
		sum of B*value	2.42	
		Plus intercept=Sum	-2.68	

The probability of this cut-point is .41, therefore the Log OR p/1 – p = .41/1-.41, thus the cut-point is −0.16. (If the number yielded from the equation is above this value, the patient should be referred for prostate biopsy). The patient is a 60 year old, Caucasian male with a recent PSA test value of 4.3 ng/dL. In addition, he had additional lab work to include a HGB, RBC count, MCV, Creatinine, and a negative test for Hematuria. Utilizing method 2, cut-point 1, the Patient’s PBCDR total sum is -2.68, which is less than -0.16. Therefore, the recommendation is no biopsy for the patient.

Patient’s PBCDR Total Sum –2.68 <–0.16. Recommendation? No Biopsy for patient.

## Discussion

The persistent inability to differentiate between indolent and aggressive PC has been one of the major limitations of PSA PC screening. To our knowledge, evaluating combinations of laboratory biomarkers, used concomitantly with an elevated PSA, has not been researched as a PSA augmentation strategy. Therefore, the key focus of this study was to determine if the laboratory biomarkers under evaluation were related to more advanced/aggressive PC stages. Our study found that HGB (OR=1.42 95% CI 1.27-1.59), RBC count (OR=2.52 95% CI 1.67-3.78), PSA (OR=1.04 95% CI 1.03-1.05), serum creatinine (OR=1.55 95% CI 1.12-2.15), and ‘Black” ethnicity (OR=1.88 95% CI 1.25-2.85) were significantly related to the PC group (method 1 PC stages I-IV). RBC count (p < 0.0001), HGB (p < 0.0001) and creatinine (p < 0.05) demonstrated increased PC risk with a 1 unit negative change in their value; while age (p < 0.005), PSA value (p < 0.005), and MCV level (p < 0.0001) demonstrated increased PC risk with 1 unit positive increase in their respected values (consistent with what one would expect). Analysis method 2 (PC stage I in comparison group) demonstrated HGB (OR 1.47 95% CI 1.31, 1.61), RBC count (OR 2.15 95% CI 1.43, 3.23), serum creatinine (OR 1.83 95% CI 1.18, 2.85), PSA (OR 1.033 95% CI 1.02, 1.05), MCV (OR 1.05 95% CI 1.02, 1.08), and age (OR 1.018 95% CI 1.01, 1.04) were significantly related to PC stages II-IV. ROC curves were compared to address whether the addition of these significant lab biomarkers would improve PC prediction when compared to PSA alone. The AUC increased from: **Primary Analysis** PSA alone 0.59, (95% CI 0.55, 0.61) to PBCDR best fit model 0.68, (95% CI 0.65, 0.71); **Secondary Analysis** PSA alone 0.63, (95% CI 0.58, 0.66), to PBCDR best fit model 0.72, (95% CI 0.68, 0.75). The differences between the models are statistically significant. In addition to the ROC curve, the validity (sensitivity/specificity) and positive/negative predictive values were determined. For the PSA only model, one can only determine the positive predictive value, given patients with a PSA less than 4 ng/dL are not routinely forwarded for prostate biopsy. The PPV of PSA alone (> = 4 ng/dL) decreased from 44.7% (method 1) to 20.6% (method 2). This indicates PSA is less effective as a tool for identifying the more clinically relevant PC (stages II-IV). Conversely, the PPV of the PBCDR method 2 (cut point 1) model was 55.3%, with a NPV of 81.9%, and specificity of 96.2%. These values demonstrate the PBCDR yielded significantly higher validity and predictive scores than that of PSA alone. In alignment with our hypothesis that there exists a gradient of change between the significant lab biomarkers and increasing levels of PC; HGB, RBC, PSA and Albumin demonstrated a significant gradient.

One important potential benefit of the PBCDR model is a reduction in prostate biopsies. In our study population, the PPV of PSA alone (in identifying stage II-IV PC) was 20.6% (284/1378). The PBCDR model (Method 2, cut-point 1), yielded a positive predictive value of 55.3% (52/94), a specificity of 96.2%, and sensitivity of 18.3%. If this particular PBCDR model was employed (as opposed to PSA alone), the number of unnecessary biopsies (those that did not identify PC stage II-IV) would have been reduced from 1,092 to 92. Moreover, with a specificity of 96.2%, the PBCDR model would correctly identify greater than 9 out of every 10 men who do not have PC. In 1998, the JH VA performed 1,610 biopsies. If one were to apply the PBCDR (comparing PC stage II-IV vs. stage I PC, PIN, BPH, and prostatitis) to the 1,378 subjects within this study, 52 of the 92 total biopsies (PPV= 55.3%) would have been positive for PC versus the PSA alone (PPV 44.7%). In addition, if the PBCDR would have been employed, approximately 1,052 negative biopsies would not have been performed. This would have resulted in a substantial decrease in cost for the VA, as well as reduced anxiety for the patient, and a reduced risk of biopsy morbidity (given the biopsies would have never been performed).

Our study was consistent with previous published reports in many ways. The proportion of PC that was localized to the prostate is consistent with the screening stage shift phenomenon (increased amounts of pre-clinical disease are detected), with Stage I comprising 53.9% of all PC cases; Stage II 35.7%; Stage III 7.8%; and Stage IV 2.59% (data not shown).

Secondly, all laboratory biomarkers demonstrated ‘movement’ in the direction away from the ‘normal’ value that is consistent with previous literature and is biologically plausible.^15^ For instance, the mean PSA value increased from 7.67 (prostatitis) to 44.03 (stage IV PC). Multiple published studies have reported that a low hemoglobin value is an independent risk factor for poor survival outcomes in patients with hormone refractory PC [11]. In addition; the correlation between PC and hematologic disorders has been long recognized for its clinical significance, with anemia a frequent clinical manifestation of advancing PC [9,11]. In this study, the laboratory parameters HGB, RBC count, and MCV (all indicators of hematologic state) demonstrated values below their normal reference range in patients with clinically relevant PC (stage II-IV). When comparing the subjects with histologically confirmed prostatitis to patients with histologically confirmed stage III PC, the difference becomes evident. HGB decreased from 14.27 to 12.59 (p < 0.05), RBC count decreased from 4.67 to 4.58 (p < 0.05). Lastly, there was an 83% increase in the risk of PC for African American (AA) men when compared to Caucasian men, which is consistent with AA ethnicity as being a major PC risk factor [8].

Our study has limitations. Men followed by the VA may be subjected to a less or more stringent screening with respect to PC than are other men in the general U.S. population and therefore may not be representative to non-VA healthcare system patient populations. In addition, certain laboratory biomarkers had incomplete data which lead to the exclusion for analysis and interpretation. Although clinicians often obtain a complete blood count (CBC), basic metabolic panel (BMP), and urinalysis (UA) at the time of PSA screening, certain assays (e.g. PT/aPTT, and Folate) are not routinely included in these panels. Both PT and aPTT are used to evaluate the coagulation system, with increasing levels of both being an independent predictor of disseminated intravascular coagulation (a systemic condition seen occasionally with metastatic PC) [12]. A high plasma levels of Folate has been previously reported as both protective and as a risk factor for PC development [13]. Given the plausible links to PC, they warrant further investigation.

Although each prostate biopsy was evaluated by two or more trained pathologists, the possibility that misclassification of disease status (i.e. patients who have PC were classified as ‘no PC’) does exist. Given that the outcome variable (PC yes or no) is determined by the results of a prostate biopsy, and that the biopsies themselves are a sampling of the entire prostate, there is a chance that the biopsy did not contain cancerous cells, yet the prostate itself does. However it is unlikely that an individual would be categorized as having PC if the carcinoma was not present on histological sample. It is more likely that a patient with PC is misclassified as not having PC, which would drive the results towards the Null hypothesis.

Other variables were not available for analysis. Information on PC family history was lacking in patient notes; and family history on any medical condition was available in less than 50% of the study participants. In addition, tobacco, alcohol use, and socio-economic status were unavailable for analysis.

A case–control design was utilized in this study. This type of design was employed as PC cases and suitable comparison subjects were identified and compared with respect to their lab values and prior exposures. While this design provided greater statistical efficiency, the potential for uncontrolled confounding and selection bias exists. Although the PBCDR tool can be used with all ages and PSA values, the lack of prostate biopsies in patients with PSA values below 4.0 ng/dl limits evaluating the effectiveness of the PBCDR in this important group.

Despite these limitations, our study has important strengths. This study was completed on a robust sample population (1,378 subjects, of which 616 had PC), and this high proportion of cases increases overall study power and statistical efficiency. This increased study power afforded us the opportunity to stratify on key parameters, such as PC stage and ethnicity. The results are biologically plausible and consistent with existing knowledge. It is accepted that there is a relationship between PC and systemic diseases that occur in the presence of both local and metastatic spread of PC [9]. Moreover, it has been demonstrated that the specified laboratory biomarkers evaluated in this study are highly correlated with the systemic diseases related to PC spread [11].

Men with PC have been described as falling into one of four groups, and screening can only really benefit one. The first group consists of men with normally progressing disease that is identified clinically; the second group includes men with PC that advances very rapidly. For the above two groups, screening is of little benefit. The third group contains men with screen-detected PC that would have never advanced to clinically relevant disease; therefore they are exposed to unnecessary procedures and treatments. Lastly, group four contains asymptomatic men who have PC identified through screening and receive beneficial outcomes that otherwise would have been deprived if not for the screening [8]. One difficulty in PC screening is identifying group 4 relative to group 3. The results of this study suggest that the PBCDR might improve our ability to detect PC while also decreasing the number of prostate biopsies. Moreover, the PBCDR seems to be more accurate with PC stages II-IV, providing a novel, simple to implement, inexpensive tool that has the potential to separate more severe PC from indolent PC. This has important implications, especially if PSA is used as a cost effective PC screening program. The overlap of PSA values in men with PC and non-cancerous prostate conditions has been well documented. A thorough physical exam with DRE is the mainstay of PC detection, and a patient’s life expectancy and co-morbidities should be considered when developing a unique treatment course of action. However this study provides a glimpse of the potential benefit that these additional lab parameters can provide.

In conclusion, our study results suggest that evaluating certain biomarkers in conjunction with PSA may improve PC prediction prior to biopsy. Moreover, the biomarkers may be more helpful in detecting clinically relevant PC. Follow-up studies should begin with replicating this study on different U.S. VA patient populations, involving multiple practices, capturing PC family history, and evaluating other biomarkers (such as PSA values lower than 4.0 ng/dl, PT/aPTT and folate) that may assist in improving PC screening efficiency.

## Competing interests

The authors declare that they have no competing interests.

## Authors’ contribution

OTH contributed to the conception, design, analysis, and writing of the manuscript. TJM contributed to the conception, design, and analysis of the manuscript. SWS contributed to the conception, design, and analysis of the manuscript. PRF contributed to the conception, design, and data acquisition. All authors contributed to editing the manuscript and have given final approval of the version to be published.

## Pre-publication history

The pre-publication history for this paper can be accessed here:

http://www.biomedcentral.com/1471-2490/13/6/prepub
